# Prognostic value of the platelet-neutrophil-monocyte-lymphocyte ratio in patients with non-metastatic renal cell carcinoma who underwent nephrectomy

**DOI:** 10.1186/s12885-025-14418-z

**Published:** 2025-06-02

**Authors:** Dan Chen, Yaxiong Tang, Bin Zhang

**Affiliations:** 1 Department of Urology, Chengdu Seventh People’s Hospital, Affiliated Cancer Hospital of Chengdu Medical College, Chengdu, 610041 China; 2 Department of Urology, Institute of Urology, West China Hospital, Sichuan University, Chengdu, 610041 China

**Keywords:** Renal cell carcinoma, Systemic inflammation, Nephrectomy, Disease-free survival, Overall survival

## Abstract

**Background:**

Various systemic inflammation indices have emerged as prognostic markers for renal cell carcinoma (RCC); however, these indices have not been comprehensively integrated. In this study, we propose a novel systemic inflammation indice, the platelet-neutrophil-monocyte-lymphocyte ratio (PNMLR), aimed at more accurately assessing survival outcomes of patients with non-metastatic RCC.

**Patients and methods:**

We conducted a retrospective analysis of non-metastatic RCC patients who underwent nephrectomy between 2009 and 2013. Restricted cubic splines (RCS) were used to observe the relationship between PNMLR and disease-free survival (DFS) as well as overall survival (OS). Receiver operating characteristic curve and the Maximally Selected Log-Rank Statistic were employed to determine the optimal cutoff value of PNMLR. Patients were then divided into two groups based on the determined cutoff values and propensity score matching (PSM) was performed to balance baseline characteristics. After that, Kaplan–Meier curves and cox regression models were utilized to evaluate DFS and OS. Finally, the concordance index (c-index) of PLNMR (before PSM) in predicting DFS and OS was calculated and compared with other systemic inflammation indices.

**Results:**

A total of 1163 patients were included. RCS showed a significant association between PNMLR and DFS as well as OS (both *p* < 0.001). The optimized PNMLR cutoff was 168. Patients with higher PNMLR exhibited larger tumor size (OR = 1.16, *p* = 0.028), higher Fuhrman grade (HR = 1.59, *p* = 0.001), and advanced pT stage (HR = 1.88, *p* = 0.003). After PSM, elevated PNMLR was associated with poorer DFS (HR = 1.56, *p* = 0.011) and OS (HR = 1.75, *p* = 0.004). The c-index of PNMLR for DFS and OS were 0.643 (95%CI, 0.596–0.689) and 0.669 (95%CI, 0.611–0.708) respectively, suggesting competitive predictive performance compared to other systemic inflammation indices.

**Conclusions:**

PNMLR is a promising prognostic marker for non-metastatic RCC. However, its moderate discriminative ability suggests that PNMLR should be used in conjunction with other established clinical parameters. Further validation, particularly in independent, contemporary external cohorts, is essential to fully harness its clinical utility.

## Introduction

Renal cell carcinoma (RCC) is one of the most common malignancies and its incidence has been steadily increasing worldwide over the past few decades [1]. The management of RCC involves various treatment modalities, including surgery, targeted therapies, and immunotherapy, with nephrectomy being the primary treatment for localized disease [2, 3]. Prognosis is one of the most important clinical concerns for patients and physicians. Although surgical resection remains the cornerstone of treatment for non-metastatic RCC, a subset of patients still experience disease recurrence or progression, highlighting the need for improved preoperative risk stratification tools [2–4].

In recent years, growing attention has been focused on the role of the immune system in cancer development and progression. Studies have shown that platelets [5], neutrophils [6], and monocytes [7] promote cancer progression through various mechanisms. Conversely, lymphocytes play a crucial role in antitumor immunity [8]. As a result of these discoveries, several systemic inflammation indices, including neutrophil-to-lymphocyte ratio (NLR) [9], platelet-to-lymphocyte ratio (PLR) [10], monocyte-to-lymphocyte ratio (MLR) [11], systemic immune-inflammation index (SII) [12], and systemic inflammation response index (SIRI) [13], have been explored and found to correlate with prognosis in a variety of cancers, including RCC. These indices provide valuable insights into the tumor immune microenvironment, thereby influencing tumor progression and patient outcomes. Considering the essential role of these four components (neutrophils, platelets, monocytes, and lymphocytes) in tumor immune responses, integrating them into a comprehensive systemic inflammation indice may provide a more accurate assessment of the tumor immune status. However, the comprehensive impact of neutrophils, platelets, monocytes, and lymphocytes on predicting prognosis in RCC patients remains inadequately studied.

Therefore, in this study, we attempted to develop a novel systemic inflammation indice that integrates these four components, the platelet-neutrophil-monocyte-lymphocyte ratio (PNMLR), and validate its prognostic value in patients with non-metastatic RCC who underwent nephrectomy.

## Patients and methods

### Patients and clinical variables

After obtaining approval from the ethics committee of West China Hospital, Sichuan University (2023–463), we conducted a retrospective review of medical records of patients with non-metastatic RCC who underwent nephrectomy between 2009 and 2013. As this study was retrospective, did not involve potential harm to participants, and did not involve identifiable patient information, this study was exempted from written informed consent by the Ethics Committee of West China Hospital, Sichuan University. All methods were performed in accordance with the Declaration of Helsinki.

Follow-up was performed through telephone calls or outpatient visits. Patients with incomplete clinical data were excluded from the study. Clinical data, including patient age, gender, smoking history, comorbidities (hypertension and diabetes), eastern cooperative oncology group (ECOG) status, surgical approach, tumor laterality, tumor size, pathological T (pT) stage, histological type, and Fuhrman grade, were extracted from medical records. All patients underwent blood routine examination within one week before nephrectomy, and when multiple preoperative tests were available, the closest data to the surgery date were used. All lesions were unilateral, and the ECOG status was < 2 for all patients. pT stage was categorized into localized (T1-T2) and locally advanced (T3-T4), while histological subtypes were classified into clear cell RCC (ccRCC) and non-ccRCC. Fuhrman grade was divided into low grade (Grade 1–2) and high grade (Grade 3–4). Based on preoperative blood test results, the PNMLR was calculated using the following formula: platelet count (× 10^9/L) × neutrophil count (× 10^9/L) × monocyte count (× 10^9/L)/lymphocyte count (× 10^9/L).

### Statistical analysis

Restricted cubic splines (RCS) were first used to observe the relationship between the level of PNMLR and DFS as well as OS. Our primary interest was DFS, receiver operating characteristic (ROC) curve and the Maximally Selected Log-Rank Statistic were used to determine the optimal cut-off value that resulted in the most significant DFS differences between the two groups. In the ROC curve, Youden index was used to determine the optimal cut-off value, while in the Maximally Selected Log-Rank Statistic, the cut-off value that yielded the largest standardized log-rank statistic was considered optimal. After determining the cutoff value, patients were stratified into two groups: those with values less than or equal to the cutoff, and those with values greater than the cutoff. Subsequently, clinical and pathological characteristics between the two groups were compared using chi-square tests and Mann–Whitney tests as appropriate. The relationship between clinical characteristics and PNMLR was analyzed using logistic regression, with significant variables from univariate logistic regression included in the subsequent multivariate logistic regression. Kaplan–Meier curves were used to estimate DFS and OS, with the log-rank test determining the significance of survival differences between the groups. Propensity score matching (PSM) was then performed in a one-to-one ratio to balance the baseline characteristics and eliminate the influence of baseline differences on survival. After PSM, Kaplan–Meier curves were then used again to estimate DFS and OS, with the log-rank test determining the significance of survival differences between the matched groups. Univariate Cox regression was then used to determine potential independent prognostic factors, and significant variables from univariate Cox regression were included in the subsequent multivariate Cox regression to determine their prognostic value. Finally, after confirming PNMLR as an independent predictor of DFS and OS, we calculated the concordance index of continuous PNMLR before PSM in predicting DFS and OS, and compared it with other systemic inflammation indices, including NLR, PLR, MLR, SII and SIRI. All tests were two-sided, and a *p*-value < 0.05 was considered statistically significant.

## Results

### Cutoff value

**Fig. 1:**
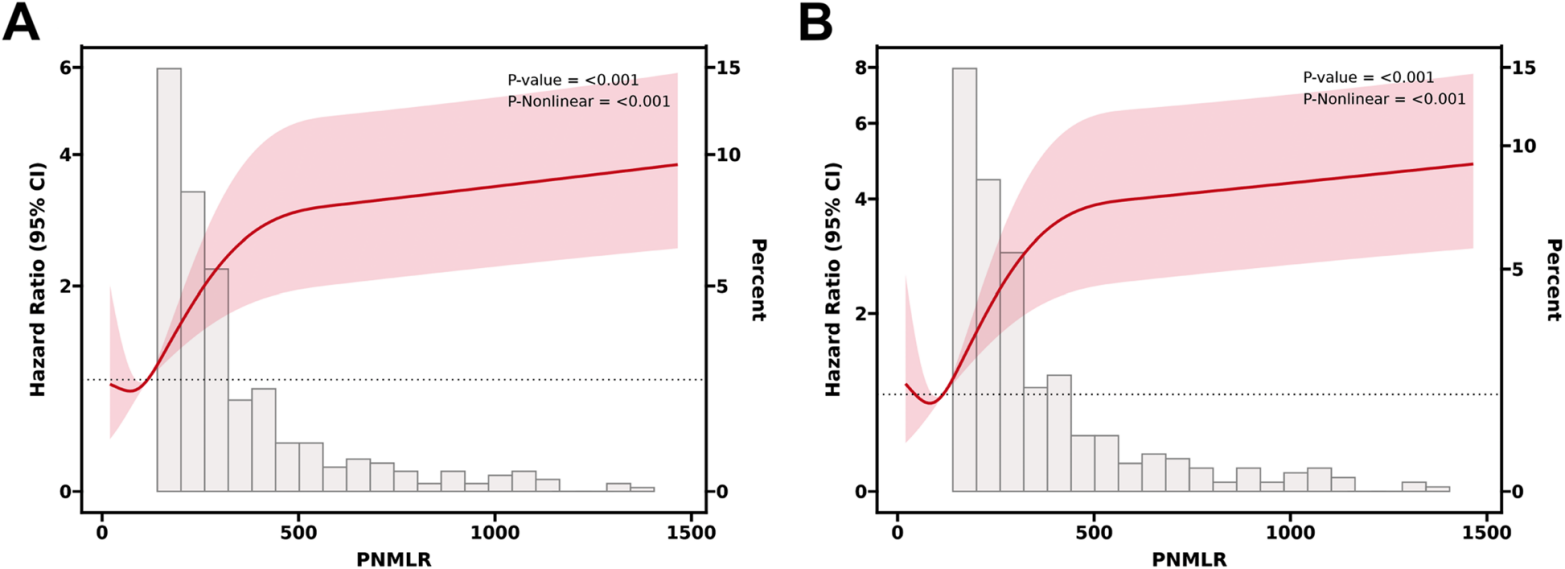
Restricted cubic splines reveal the relationship between Platelet-Neutrophil-Monocyte-Lymphocyte ratio and disease-free survival (**A**) as well as overall survival (**B**)

**Fig. 2:**
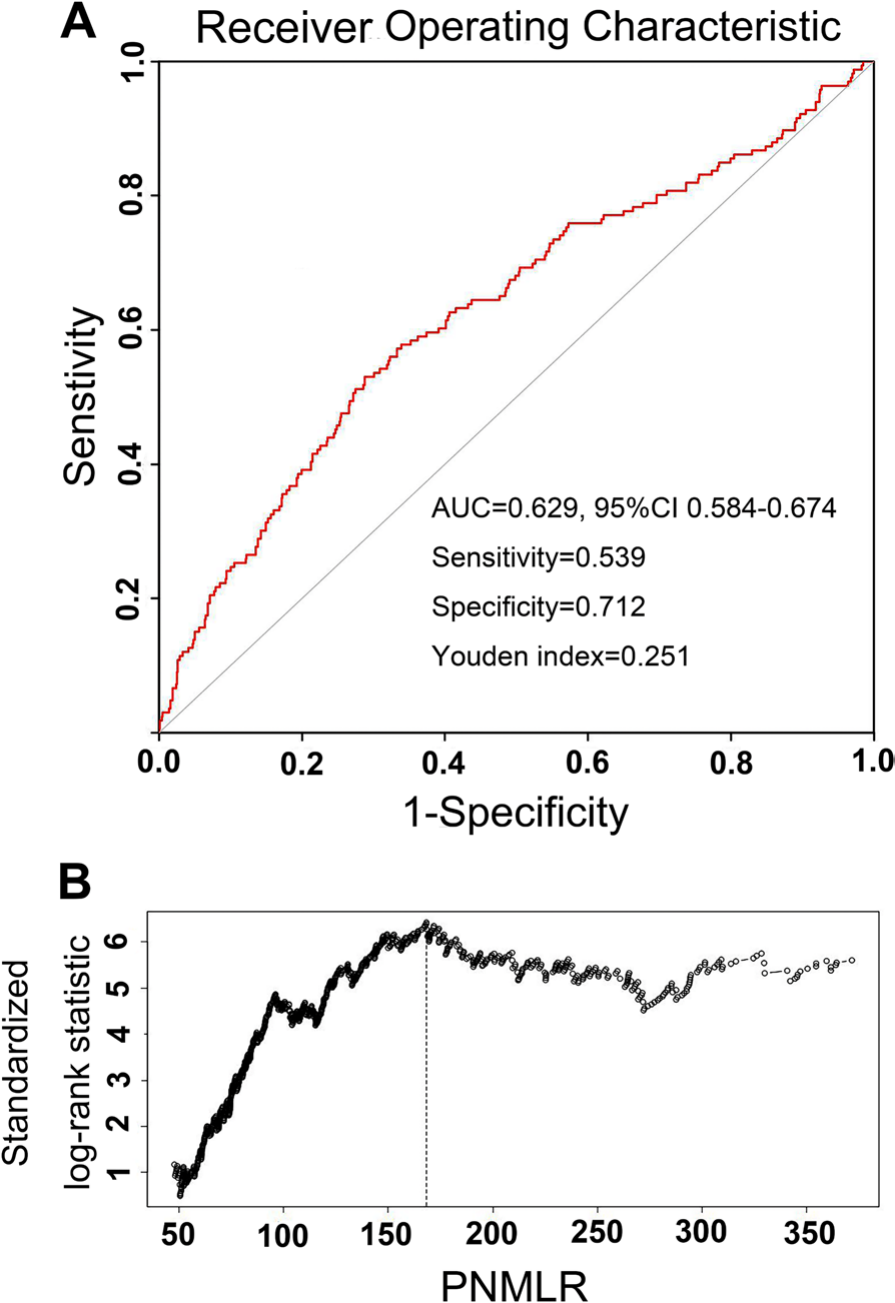
The receiver operating curve and maximally selected log-rank statistic determined the optimal cutoff values for Platelet-Neutrophil-Monocyte-Lymphocyte ratio

As shown by RCS, there was a significant negative correlation between PNMLR levels and DFS (Fig. 1A, *p* < 0.001) as well as OS (Fig. 1B, *p* < 0.001). In the ROC analysis, a PNMLR value of 168.40 yielded the maximum Youden index (Fig. 2A), while a PNMLR of 168.24 corresponded to the largest standardized log-rank statistic (Fig. 2B). Based on these findings, we pragmatically set the optimal PNMLR cutoff at 168, subsequently categorizing patients into two groups: PNMLR <  = 168 and PNMLR > 168.

### Baseline characteristics of patients before and after PSM

A total of 1163 patients were included in the study, with a median follow-up of 80 months (interquartile range [IQR], 60–90 months). The median age of the included patients was 55 years (IQR, 45–65 years), and the median tumor size was 4.4 cm (IQR, 3.0–6.0 cm). The majority of patients were male (61.3%), had an ECOG status of 0 (68.9%), localized tumors (90.6%), low-grade tumors (55.5%), and the histological subtype was predominantly ccRCC (91.7%). Radical nephrectomy was performed in 69.3% of patients. Additionally, among the participants, 330 individuals (28.4%) had a history of smoking, 257 individuals (22.1%) had hypertension, and 173 individuals (14.9%) had diabetes.

Utilizing the optimized cutoff value of 168, a total of 788 individuals (67.8%) exhibited a PNMLR <  = 168, whereas 375 individuals (32.2%) displayed a PNMLR > 168. Prior to PSM, noticeable distinctions were observed in diverse characteristics between the two cohorts. Specifically, patients with a PNMLR <  = 168 demonstrated a diminished proportion of males (58.6% vs 66.9%, *p* = 0.007), a reduced prevalence of smoking history (26.3% vs 32.8%, *p* = 0.021), and a lower incidence of hypertension (19.8% vs 26.9%, *p* = 0.006) and diabetes (12.8% vs 19.2%, *p* = 0.004) in comparison to those individuals with a PNMLR > 168. Furthermore, patients with a PNMLR <  = 168 demonstrated smaller tumor volumes (median size, 4.0 vs 5.0 cm, *p* < 0.001), a reduced proportion of local advanced tumors (6.8% vs 14.7%, *p* < 0.001), a lower frequency of high-grade tumors (40.0% vs 53.9%, *p* < 0.001), and a higher propensity to undergo partial nephrectomy (33.6% vs 24.5%, *p* = 0.002).

Following one-to-one PSM, 361 individuals each from the PNMLR <  = 168 group and the PNMLR > 168 group were included, and no statistically significant differences were observed between the two groups in any of the baseline characteristics (all *p* > 0.05). The baseline characteristics of patients before and after PSM are presented in Table 1.

**Table 1:** Patient clinical characteristics before and after propensity score matching

Variables	Before PSM				After PSM		
	Total, No. (%)	PNMLR < = 168^a^	PNMLR > 168^a^	*p*-value	PNMLR < = 168^a^	PNMLR > 168^a^	*p*-value
Age, median (IQR)	55 (45, 65)	54.5 (45, 64)	57 (45, 65)	0.358	56 (45, 66)	57 (45, 65)	0.808
Sex				0.007			0.58
Male	713 (61.3%)	462 (58.6%)	251 (66.9%)		245 (67.9%)	238 (65.9%)	
Female	450 (38.7%)	326 (41.4%)	124 (33.1%)		116 (32.1%)	123 (34.1%)	
Smoking history				0.021			0.431
No	833 (71.6%)	581 (73.7%)	252 (67.2%)		234 (64.8%)	244 (67.6%)	
Yes	330 (28.4%)	207 (26.3%)	123 (32.8%)		127 (35.2%)	117 (32.4%)	
Hypertension				0.006			0.738
No	906 (77.9%)	632 (80.2%)	274 (73.1%)		261 (72.3%)	265 (73.4%)	
Yes	257 (22.1%)	156 (19.8%)	101 (26.9%)		100 (27.7%)	96 (26.6%)	
Diabetes mellitus				0.004			0.627
No	990 (85.1%)	687 (87.2%)	303 (80.8%)		299 (82.8%)	294 (81.4%)	
Yes	173 (14.9%)	101 (12.8%)	72 (19.2%)		62 (17.2%)	67 (18.6%)	
ECOG status				0.072			0.874
ECOG 0	801 (68.9%)	556 (70.6%)	245 (65.3%)		243 (67.3%)	241 (66.8%)	
ECOG 1	362 (31.1%)	232 (29.4%)	130 (34.7%)		118 (32.7%)	120 (33.2%)	
Surgical method				0.002			0.734
RN	806 (69.3%)	523 (66.4%)	283 (75.5%)		265 (73.4%)	269 (74.5%)	
PN	357 (30.7%)	265 (33.6%)	92 (24.5%)		96 (26.6%)	92 (25.5%)	
Side				0.797			0.655
Left	586 (50.4%)	395 (50.1%)	191 (50.9%)		191 (52.9%)	185 (51.2%)	
Right	577 (49.6%)	393 (49.9%)	184 (49.1%)		170 (47.1%)	176 (48.8%)	
Size, median (IQR)	4.4 (3.0, 6.0)	4.0 (3.0, 5.4)	5.0 (3.5, 7.0)	< 0.001	4.7 (3.5, 6.2)	5.0 (3.5, 6.6)	0.441
pT				< 0.001			0.911
T1-T2	1054 (90.6%)	734 (93.2%)	320 (85.3%)		315 (87.3%)	316 (87.5%)	
T3-T4	109 (9.4%)	54 (6.8%)	55 (14.7%)		46 (12.7%)	45 (12.5%)	
Histology type				0.398			0.796
ccRCC	1066 (91.7%)	726 (92.1%)	340 (90.7%)		327 (90.6%)	329 (91.1%)	
Non-ccRCC	97 (8.3%)	62 (7.9%)	35 (9.3%)		34 (9.4%)	32 (8.9%)	
Fuhrman grade				< 0.001			0.602
G1-G2	646 (55.5%)	473 (60.0%)	173 (46.1%)		179 (49.6%)	172 (47.6%)	
G3-G4	517 (44.5%)	315 (40.0%)	202 (53.9%)		182 (50.4%)	189 (52.4%)	

*Abbreviations: PNMLR* platelet-neutrophil-monocyte-lymphocyte ratio, *PSM* propensity score matching, *IQR* interquartile range, *RN* radical nephrectomy, *PN* partial nephrectomy, *ECOG* Eastern Cooperative Oncology Group, *ccRCC* clear cell renal cell carcinoma

^a^The PNMLR was calculated based on the following formula: Platelet count (× 10^9/L) × Neutrophil count (× 10^9/L) × Monocyte count (× 10^9/L)/Lymphocyte count (× 10^9/L). The optimal cutoff value of 168 was determined using receiver operating characteristic (ROC) curve and maximally selected log-rank statistic. Subsequently, patients were categorized into two groups: those with a PNML ratio less than or equal to 168 and those with a PNML ratio greater than 168

### Clinicopathological features and PNMLR association

Logistic regression was employed to investigate the relationship between patients'clinicopathological features and the PNMLR. As shown in Table 2, in the univariate logistic regression analysis, variables including sex, smoking history, hypertension, diabetes, tumor size, pT stage, and Fuhrman grade were identified as potential predictors of the PNMLR (all *p* < 0.05) and subsequently included in the multivariate logistic regression. In the multivariate logistic regression analysis, it was found that patients with hypertension (OR = 1.41, 95%CI, 1.05–1.91, *p* = 0.028), diabetes (OR = 1.39, 95%CI, 1.02–1.99, *p* = 0.042), larger tumor size (Every 1 cm, OR = 1.16, 95%CI, 1.03–1.43, *p* = 0.028), local advanced disease (OR = 1.88, 95%CI, 1.26–2.87, *p* = 0.003), and high Fuhrman grade (OR = 1.59, 95%CI, 1.22–2.05, *p* = 0.001) were more likely to exhibit elevated PNMLR.

**Table 2:** Association between PNMLR and clinicopathological features

Variables	Univariate		Multivariate	
	OR (95%CI)	*p*-value	OR (95%CI)	*p*-value
Age (Every 10 years)	1.21 (0.94, 1.51)	0.138		
Sex (Female vs. Male)	0.70 (0.54, 0.91)	0.007	0.85 (0.63, 1.16)	0.246
Smoking history (Yes vs. No)	1.37 (1.05, 1.79)	0.021	1.28 (0.94, 1.79)	0.162
Hypertension (Yes vs. No)	1.49 (1.12, 1.99)	0.006	1.41 (1.05, 1.91)	0.028
Diabetes mellitus (Yes vs. No)	1.62 (1.16, 2.25)	0.004	1.39 (1.02, 1.99)	0.042
ECOG status (1 vs. 0)	1.27 (0.98, 1.65)	0.072		
Side (Right vs. Left)	0.97 (0.76, 1.24)	0.797		
Tumor size (Every 1 cm)	1.18 (109, 1.33)	0.001	1.16 (1.03, 1.43)	0.028
pT (Local advanced vs. Localized)	2.34 (1.57, 3.48)	< 0.001	1.88 (1.26, 2.87)	0.003
Histology type (Non-ccRCC vs. ccRCC)	1.21 (0.78, 1.86)	0.399		
Fuhrman grade (High vs. Low)	1.75 (1.37, 2.25)	< 0.001	1.59 (1.22, 2.05)	0.001

*Abbreviations: PNMLR* Platelet-Neutrophil-Monocyte-Lymphocyte ratio, *OR* odds ratio, *ECOG* Eastern Cooperative Oncology Group, *pT* pathological stage, *ccRCC* clear cell renal cell carcinoma

### Disease-free survival and overall survival

**Fig. 3:**
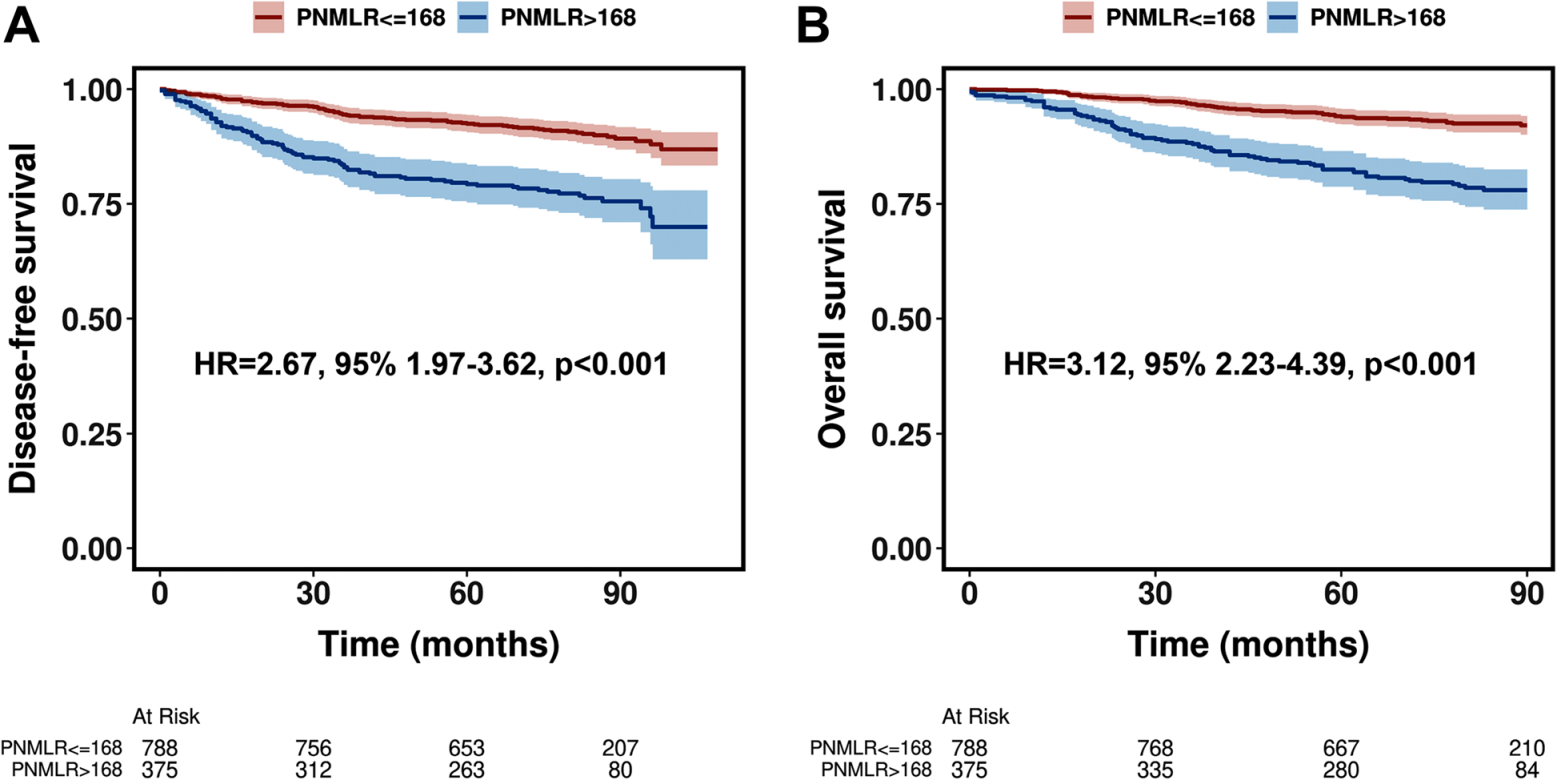
Kaplan–Meier curves illustrating disease-free survival (**A**) and overall survival (**B**) before propensity score matching

**Fig. 4:**
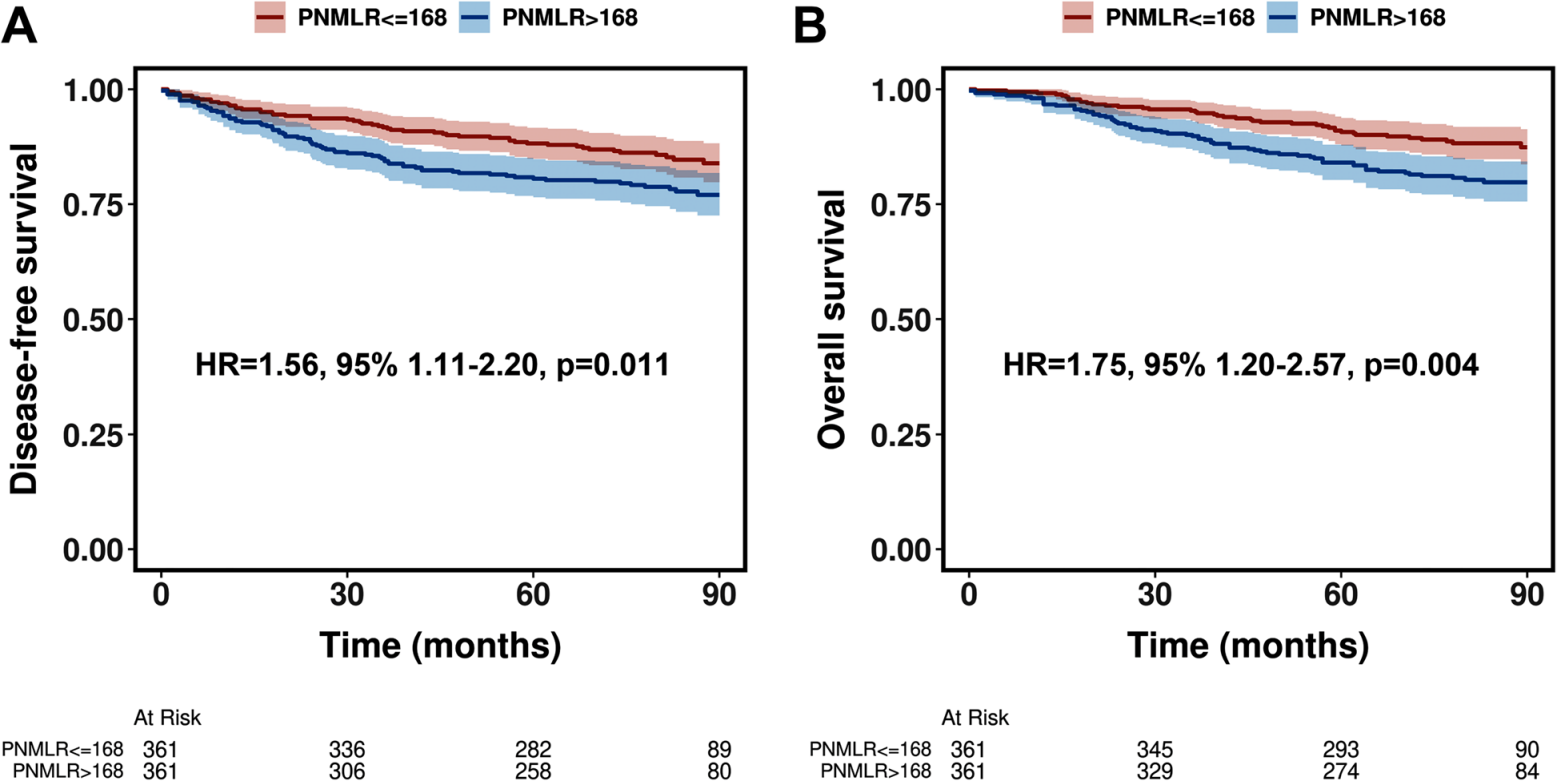
Kaplan–Meier curves illustrating disease-free survival (**A**) and overall survival (**B**) after propensity score matching

Before PSM, patients with a PNMLR > 168 demonstrated poorer DFS (HR = 2.67, 95%CI, 1.97–3.62, *p* < 0.001, Fig. 3A) and OS (HR = 3.12, 95%CI, 2.23–4.39, *p* < 0.001, Fig. 3B) compared to those with a PNMLR <  = 168. Following PSM, although the survival differences between the two groups were moderated, patients with higher PNMLR continued to exhibit significantly worse DFS (HR = 1.56, 95%CI, 1.11–2.20, *p* = 0.011, Fig. 4A) and OS (HR = 1.75, 95%CI, 1.20–2.57, *p* = 0.004, Fig. 4B).

In univariate Cox regression analysis, age, ECOG status, surgical approach, tumor size, pT stage, histological subtype, Fuhrman grade, and PNMLR were identified as potential predictors of survival (both DFS and OS) (all *p* < 0.05) and subsequently included in the multivariate Cox regression analysis. In multivariate Cox regression analysis, a high PNMLR was identified as an adverse prognostic factor for both DFS (HR = 1.61, 95%CI, 1.15–2.29, *p* = 0.005) and OS (HR = 1.81, 95%CI, 1.24–2.64, *p* = 0.003). Additionally, higher ECOG status, larger tumor size, advanced pT stage, and higher Fuhrman grade were also identified as independent adverse prognostic factors for both DFS and OS (all HR > 1, *p* < 0.05), whereas advanced age was only Identified as an independent poor prognostic factor for OS (HR = 1.32, 95%CI, 1.02–2.01, *p* = 0.042). The results of the Cox regression model are shown in Table 3.

**Table 3:** Univariate and multivariate Cox model analyses of disease-free survival and overall survival

Variables	Disease free survival				Overall survival			
	Univariate		Multivariate		Univariate		Multivariate	
	HR (95%CI)	*p*	HR (95%CI)	*p*	HR (95%CI)	*p*	HR (95%CI)	*p*
Age, year (Every 10 years)	1.78 (1.22, 2.44)	<0.001	1.12 (0.71, 1.67)	0.438	2.01 (1.53, 2.68)	<0.001	1.32 (1.02, 2.01)	0.042
Sex (Female vs. Male)	0.88 (0.61, 1.26)	0.482			0.89 (0.59, 1.32)	0.550		
Smoking history (Yes vs. No)	0.92 (0.58, 1.32)	0.582			0.96 (0.76, 1.46)	0.682		
Hypertension (Yes vs. No)	0.90 (0.44, 1.21)	0.432			0.94 (0.59, 1.38)	0.640		
Diabetes mellitus (Yes vs. No)	0.99 (0.63, 1.55)	0.973			1.17 (0.73, 1.86)	0.515		
ECOG status (1 vs. 0)	2.15 (1.53, 3.02)	<0.001	1.85 (1.31, 2.67)	0.001	2.86 (1.97, 4.16)	<0.001	2.31 (1.59, 3.48)	<0.001
Surgical method (PN vs. RN)	0.25 (0.14, 0.46)	<0.001	0.72 (0.41, 1.38)	0.301	0.20 (0.10, 0.41)	<0.001	0.52 (0.25, 1.08)	0.067
Side (Right vs. Left)	1.03 (0.74, 1.44)	0.861			1.07 (0.74, 1.55)	0.709		
Tumor size, cm (Every 1 cm)	2.01 (1.69, 2.53)	<0.001	1.74 (1.31, 2.07)	<0.001	1.89 (1.53, 2.39)	<0.001	1.68 (1.28, 1.98)	<0.001
pT (Local advanced vs. Localized)	5.43 (3.82, 7.72)	<0.001	3.51 (2.43, 5.21)	<0.001	4.80 (3.26, 7.06)	<0.001	3.08 (2.04, 4.61)	<0.001
Histology type (Non-ccRCC vs. ccRCC)	0.43 (0.19, 0.98)	0.044	0.51 (0.28, 1.16)	0.198	0.35 (0.13, 0.94)	0.037	0.48 (0.22, 1.19)	0.156
Fuhrman grade (High vs. Low)	4.12 (2.72, 6.25)	<0.001	2.82 (1.89, 4.31)	<0.001	2.94 (1.92, 4.48)	<0.001	1.89 (1.28, 2.89)	0.005
PNMLR (>168 vs. <=168)	1.56 (1.11, 2.20)	0.011	1.61 (1.15, 2.29)	0.005	1.75 (1.20, 2.57)	0.004	1.81 (1.24, 2.64)	0.003

*Abbreviations: HR* Hazard ratio, *ECOG* Eastern Cooperative Oncology Group, *PN* Partial nephrectomy, *RN* Radical nephrectomy, *pT* pathological stage, *ccRCC* clear cell renal cell carcinoma

### C-index

After confirming PNMLR as an independent predictor of DFS and OS, we calculated the concordance indices for various systemic inflammation indices predicting DFS and OS (before PSM). As shown in Table 4, PNMLR exhibited better performance in predicting DFS and OS compared to other systemic inflammation indices. For DFS, the concordance indices of NLR, PLR, MLR, SII, SIRI, and PNMLR were 0.61 (95%CI, 0.568–0.652), 0.618 (95%CI, 0.572–0.665), 0.629 (95%CI, 0.584–0.675), 0.63 (95%CI, 0.585–0.676), 0.625 (95%CI, 0.58–0.67), and 0.643 (95%CI, 0.596–0.689), respectively. For OS, the concordance indices of NLR, PLR, MLR, SII, SIRI, and PNMLR were 0.615 (95%CI, 0.569–0.661), 0.63 (95%CI, 0.58- 0.68), 0.647 (95%CI, 0.596–0.697), 0.638 (95%CI, 0.588–0.688), 0.64 (95%CI, 0.591–0.689), and 0.669 (95%CI, 0.611–0.708), respectively.

**Table 4:** The concordance index for various systemic inflammation indices predicting survival

Indices	Concordance index	
	DFS (95% CI)	OS (95% CI)
NLR	0.61 (0.568, 0.652)	0.615 (0.569, 0.661)
PLR	0.618 (0.572,0.665)	0.63 (0.58, 0.68)
MLR	0.629 (0.584,0.675)	0.647 (0.596,0.697)
SII	0.63 (0.585,0.676)	0.638 (0.588,0.688)
SIRI	0.625 (0.58, 0.67)	0.64 (0.591,0.689)
PNMLR	0.643 (0.596, 0.689)	0.669 (0.611, 0.708)

*Abbreviations: DFS* Disease-free survival, *OS* Overall survival, *NLR* Neutrophil-to-lymphocyte ratio, *PLR* Platelet-to-lymphocyte ratio, *MLR* Monocyte-to-lymphocyte ratio, *SII* Systemic immune-inflammation index, *SIRI* Systemic immune-inflammation response index, *PNMLR* Platelet-neutrophil-monocyte-lymphocyte ratio

## Discussion

Various indices derived from platelet, neutrophil, monocyte and lymphocyte counts have been widely shown to be associated with the prognosis of various cancers [14–17]. In RCC, the prognostic value of NLR [9], PLR [10], MLR [11], SII [12], and SIRI [13] has likewise been well established. Our previous work also validated and compared the prognostic performance of these derived indices [18]. The integration of these four peripheral blood cell counts may enhance prognostic accuracy; however, this combined approach has not yet been investigated in prior studies. In this study, we found that elevated PNMLR was significantly associated with adverse clinicopathological features. Specifically, higher PNMLR was correlated with larger tumor size, higher Fuhrman grade, and more advanced pT stage, highlighting its potential to reflect disease progression and aggressiveness. Moreover, elevated PNMLR emerged as an independent predictor of poorer DFS and OS. Importantly, PNMLR demonstrated higher accuracy in predicting DFS and OS compared with NLR, PLR, MLR, SII, and SIRI. However, PNMLR alone has limited prognostic value, suggesting that it should be used in conjunction with other clinical factors.

The prognostic value of these circulating blood cells may stem from their ability to partially reflect the status of tumor progression and antitumor immunity within the body.

While platelets traditionally play a crucial role in clotting and hemostasis following vascular mechanical injury, recent research has revealed their interactions with tumor cells, aiding in tumor cell hematogenous dissemination. The activation of platelets and the coagulation system shield circulating tumor cells from immune elimination, promoting their survival. Furthermore, platelets facilitate the adhesion of circulating tumor cells to endothelial cells through an array of growth factors, fostering phenotypic changes akin to mesenchymal transition, thereby promoting the formation of metastatic foci [5, 19, 20]. Similarly, the role of neutrophils in cancer remains a subject of debate. In the tumor immune microenvironment, infiltrating neutrophils display dual traits of both promoting tumor progression and antitumor activity [21]. Neutrophils exhibit direct cytotoxicity against tumor cells, inhibiting metastasis [22]. Conversely, they can facilitate tumor advancement through various mechanisms, including promoting angiogenesis, modulating other immune cells'functions, and stimulating tumor cell migration and invasion [23, 24]. Despite the dual functionality of infiltrating neutrophils, high neutrophil infiltration in the tumor immune microenvironment has been correlated with adverse prognosis in many cancers [21]. Although statistical significance is not yet reached, Galdiero et al. suggested a potential association between elevated circulating neutrophil counts and increased infiltration of tumor-promoting neutrophils in pancreatic cancer patients [24]. Circulating monocytes, once differentiated into macrophages in the extracellular space, contribute to tumor progression by inducing tumor vessel formation and suppressing antitumor immunity [25]. Lymphocytes, particularly T cells, have a pivotal role in antitumor immunity. Prior research has indicated that decreased peripheral lymphocyte counts in RCC correlate with poorer survival [26].

### Limitations

Several limitations of our study need to be acknowledged. First, the retrospective, single-center design may introduce selection bias and limit generalizability. Moreover, our cohort consisted of patients treated at a single Chinese institution from 2009 to 2013, which may not represent current treatment paradigms or diverse patient populations. This highlights the need for prospective, multicenter validation of our findings in more recent cohorts. Second, the cutoff value of PNMLR was determined using the Youden index and maximally selected log-rank statistics, but its reliability requires confirmation through independent validation cohorts. Additionally, the biological mechanism of the association between PNMLR and RCC prognosis has not been explored in this study and needs further exploration. Third, unmeasured confounders, such as concurrent inflammatory conditions, comorbidities, or genetic polymorphisms, may have influenced circulating blood cell counts and PNMLR values. Fourth, although PNMLR demonstrated a modestly higher concordance index than other inflammation-based indices, its moderate discriminative power suggests it should be interpreted alongside established clinical prognostic factors. Finally, like previous indices (e.g., NLR, PLR, MLR, SII, and SIRI), the PMNLR was constructed as a simple multiplicative combination of four cell counts to enhance its usefulness. Further use of complex weighted combinations may provide more refined predictions, but at the same time increase computational complexity and difficulty in clinical application. Therefore, future studies could explore different combinations to determine which method is most effective in specific clinical contexts.

## Conclusion

In summary, our study has established a novel prognostic index, the PNMLR, and has revealed that elevated PNMLR is associated with adverse clinical-pathological features, including larger tumor size, higher Fuhrman grade, and advanced pT stage. Notably, higher PNMLR emerged as an independent predictor of poorer DFS and OS. However, further investigations are warranted to validate these findings.

## Data Availability

The corresponding author will furnish the data upon reasonable request for the purposes of this study.

## Abbreviations

ccRCC: Clear cell renal cell carcinoma
c-index: Concordance index
DFS: Disease-free survival
ECOG: Eastern cooperative oncology group
IQR: Interquartile range
NLR: Neutrophil-to-lymphocyte ratio
MLR: Monocyte-to-lymphocyte ratio
RCC: Renal cell carcinoma
RCS: Restricted cubic splines
OS: Overall survival
PLR: Platelet-to-lymphocyte ratio
PNMLR: Platelet-neutrophil-monocyte-lymphocyte ratio
PSM: Propensity score matching
ROC: Receiver operating characteristic
SII: Systemic immune-inflammation index
SIRI: Systemic inflammation response index
